# Supplementary figures and images for: The Unfolded Protein Response Regulates Pathogenic Development of Ustilago maydis by Rok1-Dependent Inhibition of Mating-Type Signaling
Source: mBio. 2019 Dec 17;10(6):e02756-19. doi: 10.1128/mBio.02756-19 (PMC6918084; doi:10.1128/mBio.02756-19)

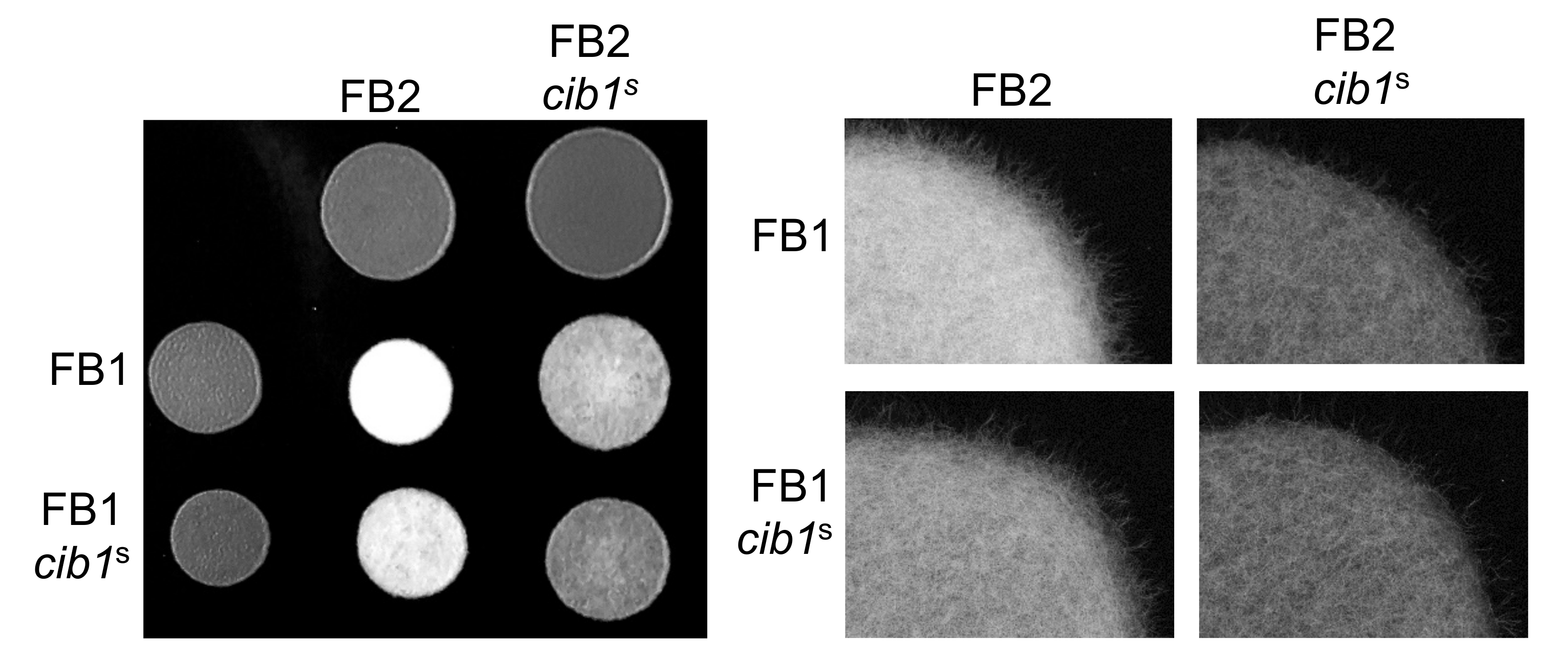

Supplement: FIG S1 [file mBio.02756-19-sf001.tif]

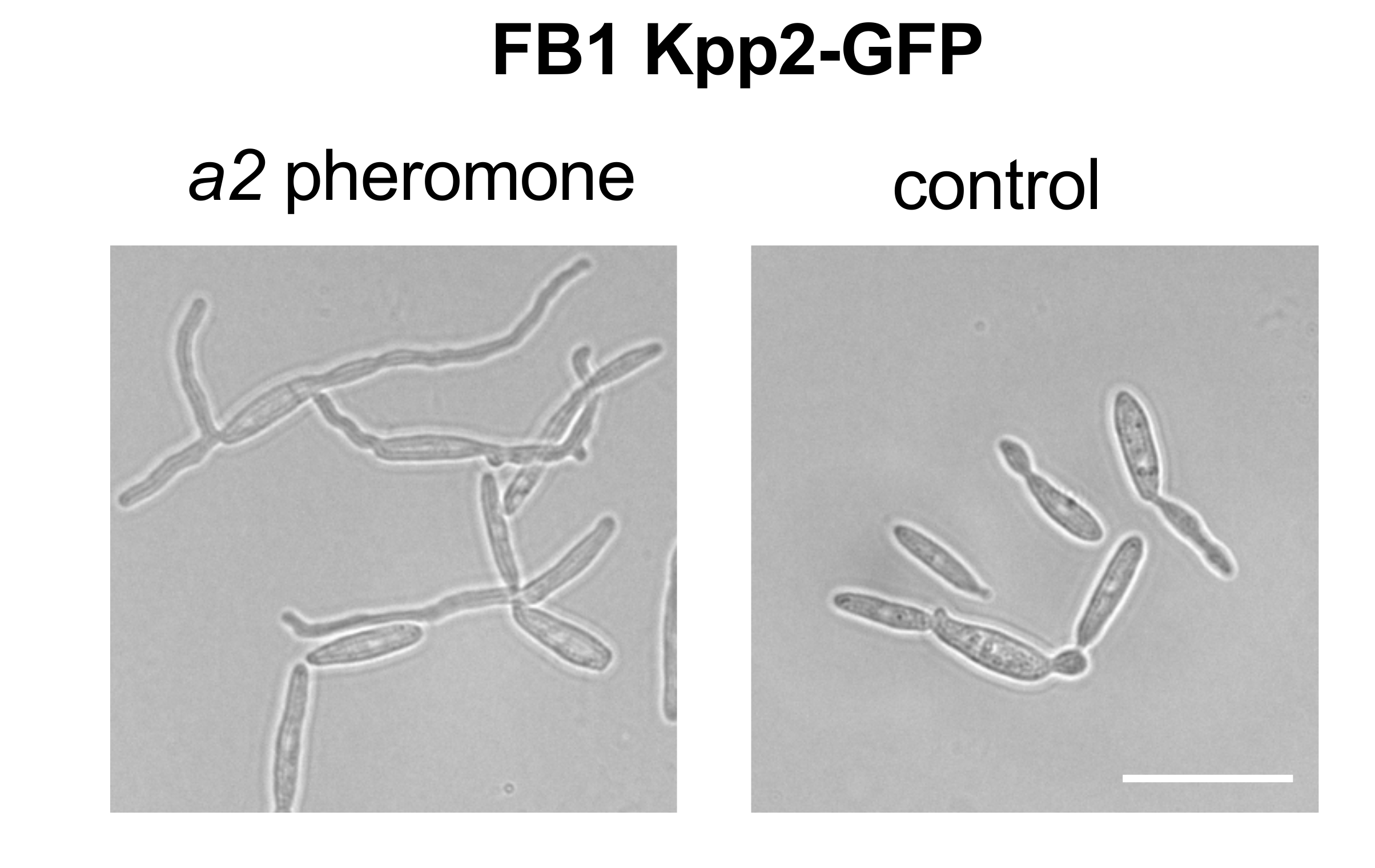

Supplement: FIG S2 [file mBio.02756-19-sf002.tif]

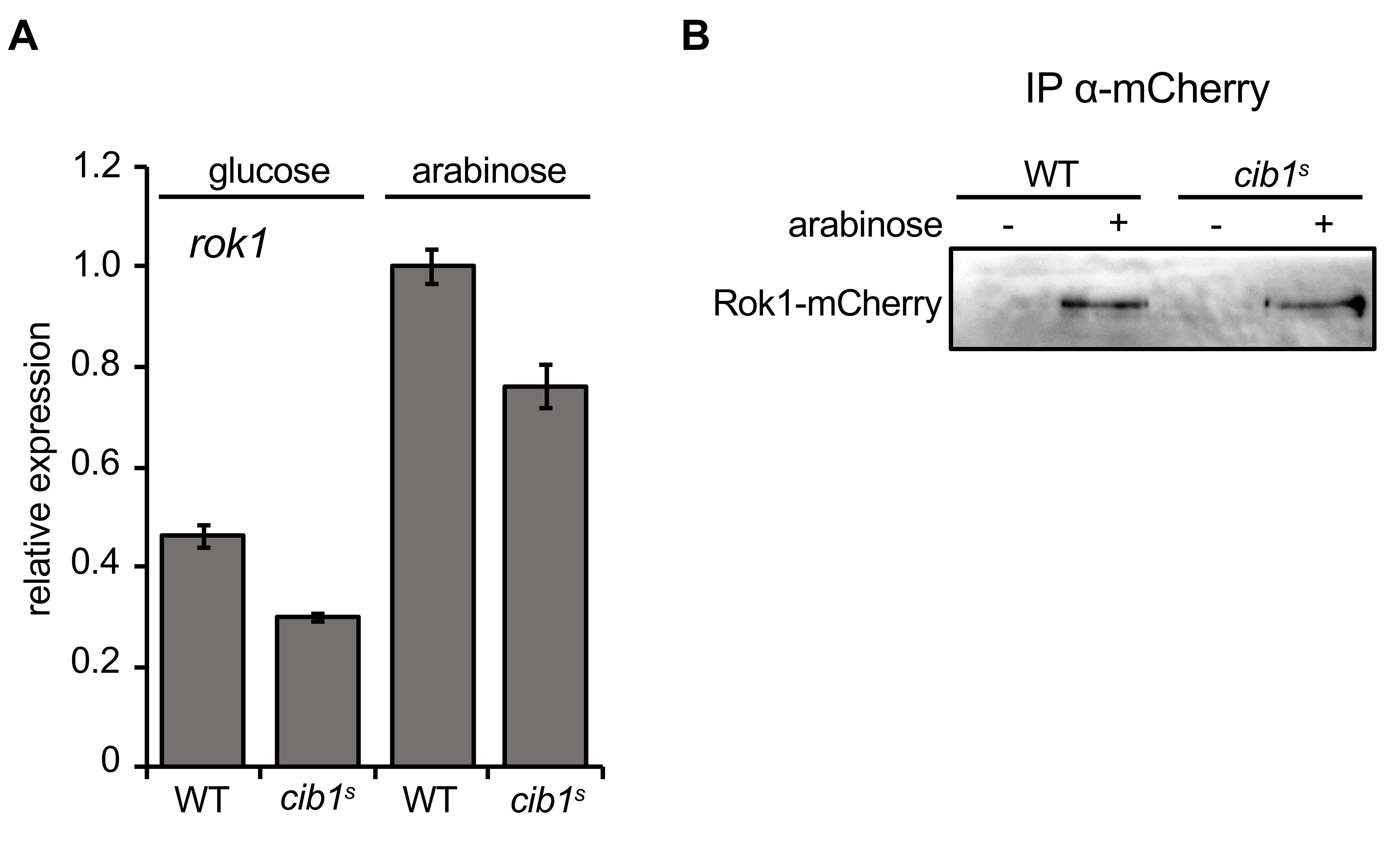

Supplement: FIG S3 [file mBio.02756-19-sf003.tif]

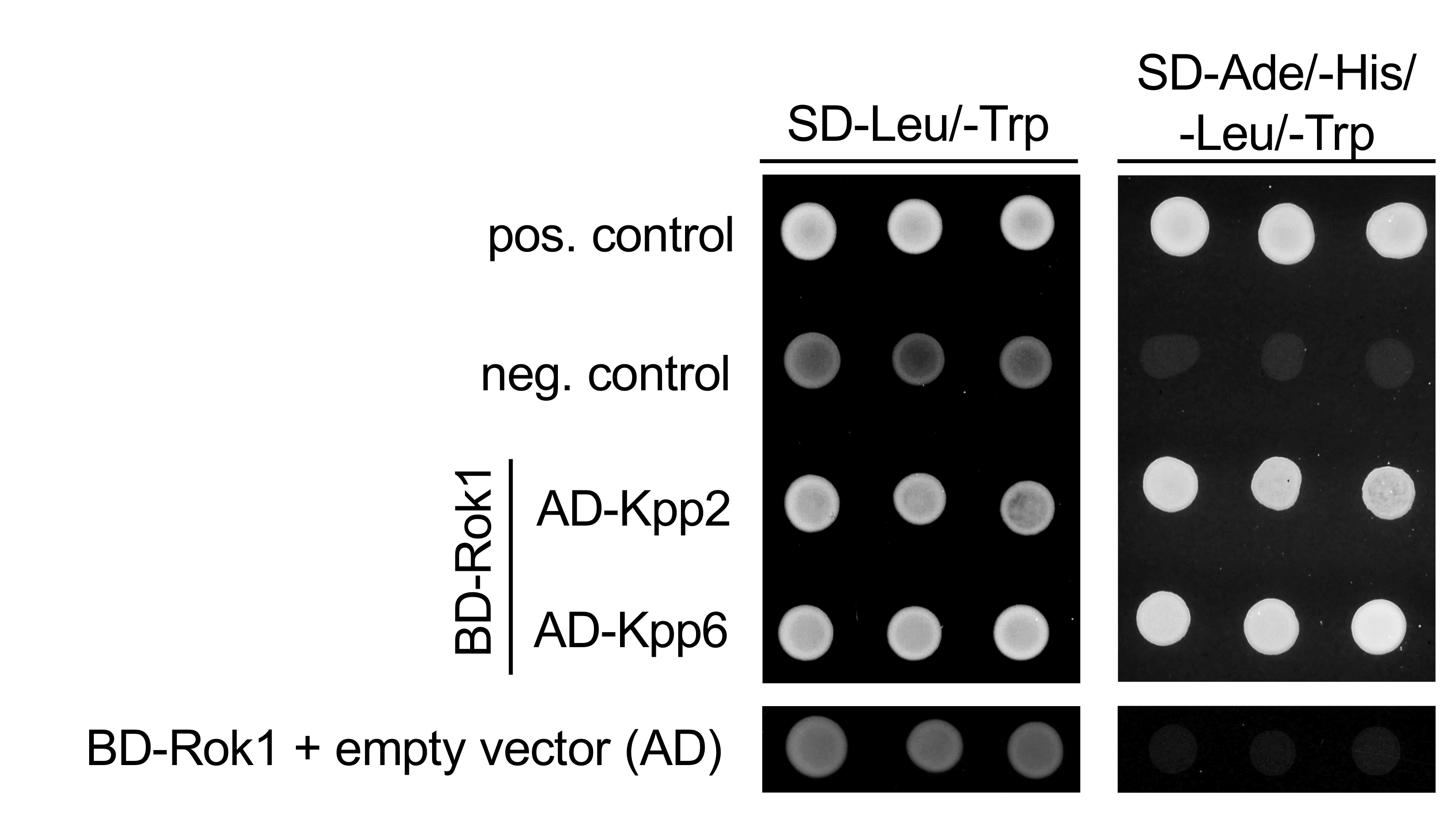

Supplement: FIG S4 [file mBio.02756-19-sf004.tif]
