# Supplementary material for: The Unfolded Protein Response Regulates Pathogenic Development of Ustilago maydis by Rok1-Dependent Inhibition of Mating-Type Signaling
Source: mBio. 2019 Dec 17;10(6):e02756-19. doi: 10.1128/mBio.02756-19 (PMC6918084; doi:10.1128/mBio.02756-19)
Supplement: TABLE S1 [file mBio.02756-19-st001.docx]

| Strain | Relevant Genotype | Reference |
| --- | --- | --- |
| FB1 | *a1 b1* | (1) |
| FB2 | *a2 b2* | (1) |
| FB1Δ*kpp2* | *a1 b1* Δ*kpp2* | (2) |
| FB2Δ*kpp2* | *a2 b2* Δ*kpp2* | (2) |
| SG200 | *a1 mfa2 bE1bW2* | (3) |
| SG200*cib1^s^* | *a1 mfa2 bE1bW2 ip*^r^ P*_cib1_:cib1*^s^ *ip*^s^ | (4) |
| SG200*cib1^s(x)^* | *a1 mfa2 bE1bW2 ip*^r^ P*_cib1_:cib1*^s^ *ip*^s^ | (4) |
| FB1*cib1^s^* (ULS1) | *a1 b1 ip*^r^ P*_cib1_:cib1*^s^ *ip*^s^ | This study |
| FB1*cib1^s(x)^* (ULS2) | *a1 b1 ip*^r^ P*_cib1_:cib1*^s^ *ip*^s^ | This study |
| FB1*fuz7^DD^* (ULS29) | *a1 b1 ip*^r^ P*_crg_:fuz7^DD^ ip*^s^ | This study |
| FB1*fuz7^DD^ cib1^s^* (ULS35) | *a1 b1 ip*^r^ P*_crg_:fuz7^DD^ ip*^s^ P*_cib1_:cib1*^s^ | This study |
| FB2 *cib1^s^* (ULS50) | *a2 b2 ip*^r^ P*_cib1_:cib1*^s^ *ip*^s^ | This study |
| ULS61 | *a1 b1 ip*^r^ P*_crg_:fuz7^DD^ ip*^s^ *kpp2-GFP* | This study |
| ULS111 | *a1 b1 ip*^r^ P*_crg_:fuz7^DD^ ip*^s^ *kpp2-GFP* P*_cib1_:cib1*^s^ | This study |
| ULS139 | *a1 b1 ip*^r^ P*_crg_:fuz7^DD^ ip*^s^ *kpp2-GFP* Δ*rok1* | This study |
| ULS142 | *a1 b1 ip*^r^ P*_crg_:fuz7^DD^ ip*^s^ *kpp2-GFP* P*_cib1_:cib1*^s^ Δ*rok1* | This study |
| ULS147 | *a1 b1* P*_kpp2_:kpp2*-GFP *ip*^r^ P*_crg_:fuz7^DD^-3xHA ip*^s^ | This study |
| ULS161 | *a1 b1* P*_kpp2_:kpp2*-GFP P*_cib1_:cib1*^s^ *ip*^r^ P*_crg_:fuz7^DD^-3xHA ip*^s^ | This study |
| ULS162 | *a1 b1 ip^r^* P*_crg_:fuz7^DD^ ip^s^ kpp2-GFP rok1-mCherry* | This study |
| ULS165 | *a1 b1 ip*^r^ P*_crg_:fuz7^DD^ ip*^s^ *kpp2-GFP* P*_cib1_:cib1*^s^ *rok1-mCherry* | This study |
| ULS169 | *a1 b1* P*_crg_:fuz7^DD^-3xHA* | This study |
| ULS302 | *a2 b2* Δ*kpp2* Δ*UMAG_12184::kpp2* | This study |
| ULS331 | *a1 b1* Δ*kpp2* Δ*UMAG_12184::kpp2* | This study |
| ULS343 | *a2 b2* Δ*kpp2* Δ*UMAG_03597::kpp2* | This study |
| ULS355 | *a1 b1* Δ*kpp2* Δ*UMAG_03597::kpp2* | This study |
| ULS367 | *a1 mfa2 bE1bW2 ip*^r^ P*_cib1_:cib1*^s^ *ip*^s^ Δ*rok1* | This study |

References

1. Banuett F, Herskowitz I. 1989. Different *a* alleles of *Ustilago maydis* are necessary for maintenance of filamentous growth but not for meiosis. Proc Natl Acad Sci 86:5878–5882.

2. Müller P, Aichinger C, Feldbrügge M, Kahmann R. 1999. The MAP kinase Kpp2 regulates mating and pathogenic development in *Ustilago maydis*. Mol Microbiol 34:1007–1017.

3. Kämper J, Kahmann R, Bölker M, Ma L-J, Brefort T, Saville BJ, Banuett F, Kronstad JW, Gold SE, Müller O, Perlin MH, Wösten HAB, de Vries R, Ruiz-Herrera J, Reynaga-Peña CG, Snetselaar K, McCann M, Pérez-Martín J, Feldbrügge M, Basse CW, Steinberg G, Ibeas JI, Holloman W, Guzman P, Farman M, Stajich JE, Sentandreu R, González-Prieto JM, Kennell JC, Molina L, Schirawski J, Mendoza-Mendoza A, Greilinger D, Münch K, Rössel N, Scherer M, Vraneš M, Ladendorf O, Vincon V, Fuchs U, Sandrock B, Meng S, Ho ECH, Cahill MJ, Boyce KJ, Klose J, Klosterman SJ, Deelstra HJ, Ortiz-Castellanos L, Li W, Sanchez-Alonso P, Schreier PH, Häuser-Hahn I, Vaupel M, Koopmann E, Friedrich G, Voss H, Schlüter T, Margolis J, Platt D, Swimmer C, Gnirke A, Chen F, Vysotskaia V, Mannhaupt G, Güldener U, Münsterkötter M, Haase D, Oesterheld M, Mewes H-W, Mauceli EW, DeCaprio D, Wade CM, Butler J, Young S, Jaffe DB, Calvo S, Nusbaum C, Galagan J, Birren BW. 2006. Insights from the genome of the biotrophic fungal plant pathogen *Ustilago maydis*. Nature 444:97–101.

4. Heimel K, Freitag J, Hampel M, Ast J, Bölker M, Kämper J. 2013. Crosstalk between the Unfolded Protein Response and Pathways That Regulate Pathogenic Development in *Ustilago maydis*. Plant Cell 25:4262–4277.
