# Supplementary material for: The Unfolded Protein Response Regulates Pathogenic Development of Ustilago maydis by Rok1-Dependent Inhibition of Mating-Type Signaling
Source: mBio. 2019 Dec 17;10(6):e02756-19. doi: 10.1128/mBio.02756-19 (PMC6918084; doi:10.1128/mBio.02756-19)
Supplement: TABLE S2 [file mBio.02756-19-st002.docx]

**Supplemental Table 2: Primers used in this study**

| **Purpose** | **Name** | **Sequence** | **Reference** |
| --- | --- | --- | --- |
| qRT-PCR | RT_eIF2b_f | ATCCCGAACAGCCCAAAC | (1) |
|  | RT_eIF2b_r | ATCGTCAACCGCAACCAC | (1) |
|  | RT-Biz1_f | CTGACCGAGGCTACTGGC | This study |
|  | RT-Biz1_r | ATCAATCGGCGGGTTGGATG | This study |
|  | RT_hdp1_f | CCGAAAGCGTCTGGGATGAG | (2) |
|  | RT_hdp1_r | GTCGTGCGTACATCGTACGG | (2) |
|  | RT_hdp2_f | GGCGCTTTGCATTGGAAC | (2) |
|  | RT_hdp2_r | AGCTTGAAGCCGATCGAC | (2) |
|  | RT_hap2-f | AGACTCGCGACAAGGCCC | This study |
|  | RT_hap2_r | CCTCCAGGACCTCTTGGC | This study |
|  | RT_rop1_f | ACTGGCTACACCACAATCGCC | This study |
|  | RT_rop1_r | TGACGCGGTGGCTGCTGTT | This study |
|  | RT_mfa1_f | ATGCTTTCGATCTTCGCTCAGAC | (1) |
|  | RT_mfa1_r | TAGCCGATGGGAGAACCGTTG | (1) |
|  | RT_pra1_f | AACCGAAGGCATCTGCACTGC | (1) |
|  | RT_pra1_r | CCCGCATGTCGATGTCAGACT | (1) |
|  | RT_prf1_f | TCGGTAGAACGAGCTGTGATG | (3) |
|  | RT_prf1_r | CTGTTGGACGATGTTGGAGTTG | (3) |
|  | RT-um05348-f | CGAGGGACGAAATCGTGGTC | This study |
|  | RT-um05348-r | CAGTCCATGAACGCTTTTGCTC | This study |
|  | RT-um10838-f | GGCTCAAGACGATCTCGTTCA | This study |
|  | RT-um10838-r | CCCGTTCCATCGATTCTGCAT | This study |
|  | RT-um06190-f | AGTATTGGGACGACTGCTCCG | This study |
|  | RT-um06190-r | ATGCTGATACCGGCGATGCC | This study |
|  | RT-um00306-f | GTGAGTGGATGGCGTTTAGCA | This study |
|  | RT-um00306-r | TTCGCTTCGTGAATGTACCATG | This study |
|  | RT_rok1_r | GAGCAGGATATCCACCTTGC | This study |
|  | RT_rok1_f | CTGTCTTCTTCGTCGTCGTC | This study |
|  | RT_rbf1_f | AGTACGAGCTACGACGGATTC | (4) |
|  | RT_rbf1_r | GGGTAGGTGTTGGACACATTC | (4) |
| Kpp2-GFP fusion | kpp2_fus_lf | CGTACAAGGTCGTCGATGTG | This study |
|  | kpp2_fus_lr | TTTGGCCGCGTTGGCCGCACGCATGATCTCGTTATAAATCAAC | This study |
|  | kpp2_fus_rf | AAAGGCCTGAGTGGCCCGAATTCGCTCCGCTCTAG |  |
|  | kpp2_fus_rr | GCACTGGCCTCAACTCTAAG |  |
| Fuz7^DD^-HA | Fuz7DD-HA_f | TATACATATGCTTTCGTCCGGTGC | This study |
|  | Fuz7DD-HA_r | TATACATATGTTAGGCATAGTCGGGCACGTCGTAGGGGTACTTCATCCCATCGGCCCAT | This study |
| Rok1-mCherry | rok1_fus_rf | TTTGGCCTGAGTGGCCACTTGAATCAATATTTTTGAGGATGCG | This study |
|  | rok1_fus_lf | GCTCAGGATCGACTCGAGG | This study |
|  | rok1_fus_lr | TTTGGCCGCGTTGGCCGCCGAGAGGCTGTCGCTGTC | This study |
| *rok1* deletion | rok1_KO_lf | CAGCTGGCACTTTGTCACTCACG | This study |
|  | rok1_KO_lr | GTGGGCCATCTAGGCCGTGTGTGGTCGAGATTTGACAGC | This study |
|  | rok1_KO_rf | CACGGCCTGAGTGGCCGGATGCGATCAACACATACAGTGAAGG | This study |
|  | rok1_KO_rr | GTTGCCAACTCTGTCGGCTATCG | This study |
| *kpp2* promoter fusion | kpp2_SfiI_for | TATAGGCCTGAGTGGCCATGTCACATGCCCACGGAC | This study |
|  | kpp2_SfiI_rev | TATAGGCCATCTAGGCCTCAACGCATGATCTCGTTATAAATC | This study |
|  | Hyg_f | TATAGGCCTAGATGGCCAGAAGTTC | This study |
|  | Hyg_r_BamHI | TATAGGATCCGAAGTTCCTATACTTTCTAGAGAATAG | This study |
|  | um12184_LB_f | CCAAGGCTAACGTGATGGATAC | This study |
|  | um12184_lf_SfiI | TATAGGCCACTCAGGCCGGTAACTTTCTGTCTTGTACAAGGT | This study |
|  | um12184_rf_BamHI | TATAGGATCCAGCAGAGTAGGAACGAGTGGT | This study |
|  | um12184_RB_r | GACTCACAGACTCGACTGTG | This study |
|  | um03597_KO_lf | GGTGCCAGAGAGGATGCAAG | This study |
|  | um03597_KO_rr | GACGGTAACAACGCGTATACC | This study |
|  | um03597_lf_SfiI | TATAGGCCACTCAGGCCGGTGCAGAGCTGACGGAAG | This study |
|  | um03597_rf_KpnI | TATAGGTACCCGTCAAGTCAACTTTACCGGTC | This study |
| Y2H | kpp2_Y2H_for | TATAGGCCATTACGGCCATGTCACATGCCCACGGAC | This study |
|  | Y2H_kpp2_rev | ATATGGCCGAGGCGGCCTCAACGCATGATCTCGTTATAAATC | This study |
|  | rok1_Y2H_for | TATAGGCCATTACGGCCATGGATTTACGCAACGCTAACCT | This study |
|  | Y2H_rok1_rev | ATATGGCCGAGGCGGCCCTACGAGAGGCTGTCGCT | This study |
|  | kpp6_Y2H_for | TATAGGCCATTACGGCCATGTCGATTGCCAATGCCTCTT | This study |
|  | Y2H_kpp6_rev | ATATGGCCGAGGCGGCCTCAACGAAGAAGCGGCTGAAAT | This study |

**References**

1. Heimel K, Scherer M, Schuler D, Kämper J. 2010. The *Ustilago maydis* Clp1 Protein Orchestrates Pheromone and *b*-Dependent Signaling Pathways to Coordinate the Cell Cycle and Pathogenic Development. Plant Cell 22:2908–2922.

2. Kellner N, Heimel K, Obhof T, Finkernagel F, Kämper J. 2014. The SPF27 Homologue Num1 Connects Splicing and Kinesin 1-Dependent Cytoplasmic Trafficking in *Ustilago maydis*. PLoS Genet 10:1–20.

3. Zarnack K, Eichhorn H, Kahmann R, Feldbrügge M. 2008. Pheromone-regulated target genes respond differentially to MAPK phosphorylation of transcription factor Prf1. Mol Microbiol 69:1041–1053.

4. Scherer M, Heimel K, Starke V, Kämper J. 2006. The Clp1 Protein Is Required for Clamp Formation and Pathogenic Development of *Ustilago maydis*. Plant Cell 18:2388–2401.
